# Psychoneurological Links Contributing to Body Mass Index and Eating Disorder Severity

**DOI:** 10.3390/nu17020296

**Published:** 2025-01-15

**Authors:** Geovanny Genaro Reivan Ortiz, Braulio Elizalde, Cristhian Tapia, Roser Granero

**Affiliations:** 1 Catholic University of Cuenca, Cuenca 010107, Ecuador; 2 Ministry of Public Health, Mental Health Area Zonal 6, Cuenca 010107, Ecuador; 3 Department of Psychobiology and Methodology, Universitat Autònoma de Barcelona, 08193 Barcelona, Spain; roser.granero@uab.cat

**Keywords:** emotion regulation, decision-making, impulsivity, anxiety, stress, eating disorders, body mass index

## Abstract

Background-objectives: Multiple dynamic interacting factors contribute to the presence and progression of eating disorders (ED). Empirical research has provided mixed findings regarding the mechanisms explaining the contribution of body mass index (BMI) to the diverse ED endophenotypes. The present study aims to evaluate the underlying processes (direct and indirect effects) contributing to BMI and ED severity, considering the contribution of multiple neuropsychological constructs. Method: Path analysis, implemented through structural equation models (SEM), was applied to a sample of *N* = 193 ED patients, men and women, aged 17 to 50 years old, and diagnosed with bulimia nervosa, night eating syndrome, binge eating disorder, and other specified feeding. Results: BMI was directly associated with ED severity level. The ED symptom level was also a mediational link into the relationship between BMI with emotion regulation strategies, decision-making capacity, stress levels, and impulsiveness. Multigroup SEM revealed invariance of the structural coefficients by sex, but differences according to the ED subtype. Conclusions: This study provides new empirical evidence on predictors of ED severity, focusing on the role of impaired decision-making and BMI. Our results could contribute to new intervention plans with techniques specifically aimed at improving emotional regulation capacity, decreasing impulsivity levels, and improving reasoning skills. Nutrition education plans may also play a key role for preventing the onset and progression of ED, helping patients understand how food affects their physical and emotional health and how to manage anxiety and fears related to food.

## 1. Introduction

Nutrition and diet are crucial elements of health. Studies have demonstrated that a balanced diet containing all essential nutrients is vital for optimal brain performance and cognitive function. Conversely, diets lacking in nutritional quality or are excessively abundant in calories have been linked to various negative health outcomes [1], including overweightness/obesity and even brain damage. Persistent alterations in nutritional habits affect brain chemistry and psychological processes, contributing to the onset and progression of eating disorders.

Malnutrition affects metabolic health through diverse biological pathways and determines the obesogenic effects of food, contributing to reduced body mass index (BMI) but also obesity [2]. BMI is a measure that relates body weight to height, and it is typically interpreted as an index of body fat and of healthy weight. Low BMI, which is categorized as underweight (less than 18.5 kg/m^2^ among adulthood population), suggests that the body may not be receiving the nutrients required for a healthy state and can lead to physical illness (such as osteoporosis, skin-hair-teeth problems, anemia, or irregular periods) and mental disease (such as depression, anxiety, or anorexia). On the other end of the scale, a high BMI is categorized as overweight (BMI higher than 25 and lower than 30) or obesity (BMI of 30 or greater), indicates excessive body fat deposits that can also impair health, increasing the likelihood of multiple health issues and severe comorbidities (such as heart disease, diabetes, hypertension, metabolic syndrome, substance abuse, depression, anxiety, and eating behavior related problems) [3,4,5,6].

Overweightness and obesity are complex multifactorial diseases, primarily stemming from an imbalance between energy intake (diet) and energy expenditure (physical activity). The eating disorders (ED) defined in the current version of the Diagnostic and Statistical Manual of Mental Disorders DSM-5 [7] more frequently associated with overweightness and obesity are bulimia nervosa (BN, characterized by episodes of overeating followed by regret and purging behaviors), night eating syndrome (NES, where individuals consume large amounts of food after dinner and may wake up at night to eat), binge eating disorder (BED, in which individuals eat an excessively large amount of food in a short period while feeling a loss of control), and other specific feeding and eating disorders (OSFED, which present incomplete or atypical symptoms of eating disorders). The global prevalence of ED is increasing for all subtypes and more affected people have sought professional help in recent decades [6].

The links between ED with BMI are complex, and it has been suggested that there are diverse mediational links that contribute, including emotional regulation strategies [2,8,9,10,11], impairing eating attitudes [12], negative mood states [6,10], and different neuropsychological processes (such as impaired decision-making [13,14,15,16,17,18,19]). Impulsivity has also been observed within some pathways, and the transdiagnostic role of this construct has been suggested [20].

Firstly, the difficulties in managing and responding to emotional experiences have been widely linked with overeating (for example, binge episodes) and purging behaviors (aimed at weight control/reduction). Interestingly, the emotion-driven model addresses overeating because of the dysfunction in emotional and cognitive processing [2]. This view holds that decision-making inherently relies on emotional processes that provide important implicit and explicit knowledge by which the individual can make fast and adaptive decisions [14]. Concretely, homeostatic control of food intake seems strongly influenced by the brain reward system [15]. Since dopamine and serotonin participate in the regulation of appetite, in dietary balance, and in food reward [16], it may be the case that impairing the functioning of these two neurotransmitters is related to decision-making, emotional eating, and addictive behavior. In addition, impaired decision-making under conditions of uncertainty has been associated with the concrete expression and maintenance of ED [14], and it has been suggested that both ED and obese individuals may share a similar incapacity to modulate reward and punishment using a long-term perspective, with their behavior guided by immediate rewards (restrictive eating, binges, and compensatory behaviors such as purges or laxatives), despite the severe long-term effects on their bodies and weight. This process could also explain the high resistance to change among ED patients.

Secondly, emotional dysregulation has been found to be associated with impulse control difficulties and decision-making deficits, two critical mechanisms in the basis of the onset and the chronic course of ED [13,17]. Concretely, high impulsivity and impaired emotion regulation capacities at a younger age have been related to the emergence of eating behavior symptoms later via suboptimal decision-making [18]. Reduced functionality of the ventromedial prefrontal cortex has been associated with worse decision-making performance among patients with ED [16].

Regarding mood states, perceived levels of anxiety and stress are powerful risk factors for overweightness/obesity and ED [6,10,11]. Epidemiological research shows that social anxiety and generalized anxiety are the most common psychiatric comorbidities in patients with ED, with rates around 50% in AN, BN, and BED. Studies have observed a relationship between neuropsychological performance and anxiety levels within samples of patients with ED [19], suggesting that: (a) executive functions could achieve a transdiagnostic process in ED and obesity, and (b) this process could provide explanations for the inability to regulate food intake and other dysfunctional eating behaviors among these patients.

But despite recognizing the associations between the multiple neuropsychological processes on the profiles of ED, few studies have analyzed the effect of these underlying mechanisms and the mediational links between these variables on the patients’ BMI and symptoms of the ED, over a broad spectrum of ED and weight. The aim of this study is to explore the pathways between emotional regulation, eating behavior style, decision-making, impulsiveness, anxiety, and perceived stress on the BMI and severity of the ED, among a sample of patients diagnosed with OSFED, BED, NES, and BN.

## 2. Materials and Methods

### 2.1. Participants

The study included a sample of patients diagnosed with BN (*n* = 42), NES (*n* = 31), BED (*n* = 37), and OSFED (*n* = 83) who consecutively attended to the psychiatry outpatient area of the Customer Care Program, Citizen “Mental Health” of the Ministry of Public Health of Ecuador. The diagnosis of the patients was made by the experienced psychiatrists in charge of the program. The inclusion criteria for this study were ages 17 to 50 years and met clinical criteria for BN, NES, BED, and OSFED according to DSM-5 criteria. Exclusion criteria were psychoactive substance dependence, significant medical or neurological disease, and severe cognitive impairment (such as Alzheimer’s disease).

The included patients met the criteria established by the DSM-5 for diagnosis at least 2 months before entering the study. Likewise, they did not report being under the effect of any medication or psychotropic drug at the arrival to the therapy unit.

### 2.2. Measures

BMI was calculated by dividing the weight (in kilograms) by the height (in meters squared). The weight was measured with a 500 KL eye-level digital Healthometer medical scale, 500 pound/485.0 kg capacity, manufactured in the USA, used to calculate BMI (BMI = weight (kg)/[height (m)]^2^.

The symptom level was measured with the Eating Disorder Inventory level (EDI-3) [20]. This is a self-report instrument used to assess psychological traits and constructs clinically related to ED. The questionnaire consists of 91 items registered in a Likert-type scale (from 0 to 5), organized in the following primary scales: drive for thinness, bulimia, body dissatisfaction, low self-esteem, personal alienation, interpersonal insecurity, interpersonal distrust, interoceptive deficits, emotional dysregulation, perfectionism, asceticism, and maturity fears. A total score in the whole questionnaire is interpreted as the ED severity level. In this work, the Cronbach’s alpha ranged between 0.83 and 0.91.

The anxiety level was assessed with the Goldberg Anxiety and Depression Scale (GADS) [21]. This self-report tool consists of 18 items (registered with a binary scale yes/no) structured in two primary scales for assessing anxiety and depression symptoms. The anxiety scale was used in the study with an adequate internal consistency in the sample (α = 0.75).

The stress level was assessed with the Perceived Stress Scale (PSS-10) [22]. This is a 10-item tool designed to assess perceived stress over the last month, based on 10 items registered through a Likert-type scale (0 = Never, 1 = Almost Never, 2 = Sometimes, 3 = Often and 4 = Very often). The Cronbach’s alpha coefficient of in the study was adequate (α = 0.79).

Emotional regulation capacities were assessed with the Emotional Regulation Questionnaire (ERQ) [23]. This is a self-report tool containing two first-order scales: cognitive reappraisal and expressive suppression. A total score is also available based on the sum of all the items. The responses are registered through a seven-point Likert scale (from 1 “totally disagree” to 7 “totally agree”). In this work the Cronbach’s alpha coefficient was α = 0.70 for the cognitive reappraisal subscale and α = 0.78 on the expressive suppression subscale.

Impulsivity level was assessed with the Plutchick Impulsivity Scale (PIS) [24,25]. This self-report scale consists of 15 Likert type items scored from 0 to 3 (never, sometimes, often, almost, and always). The aim is to obtain a measure of the control capacity for self-control, planning and action in the future, physiological behaviors, and spontaneous action. A total score calculated as the sum of the items is usually employed as a global measure of the impulsiveness levels. In this work, the Cronbach’s alpha coefficient was α = 0.70.

The decision-making capacity was assessed with the General Decision-Making Style (GDMS) [26]. This is a 25 items questionnaire (registered with a Likert scale scored from 1 = “Strongly disagree” to 5 = “Strongly agree”) developed to assess an individuals’ decision-making styles in five approaches: rational (logical approach to decisions by searching information and alternatives, and carefully thought out), intuitive (decisions based on people’s hunches or feelings), dependent (decision process based on searching advice and guidance from others), avoidant (procrastinating and avoidant decision rules), and spontaneous (quick decision process). This study analyzed a total score, calculated by summing the responses to the items. Higher values indicate a greater level of deterioration. In this work, Cronbach’s alpha coefficient in the study was α = 0.81.

### 2.3. Procedure

The study was carried out in accordance with the latest version of the Declaration of Helsinki. The Research Ethics Committee of the Catholic University of Cuenca and the Clinical Research Ethics Committee of the Ecuadorian Ministry of Public Health approved the study. Signed consent was also obtained from the participants.

Physicians and psychiatrists with high experience in the treatment of ED carried out the assessment of the patients at the baseline (at the arrival to the therapy unit). Data collection was 8 months (from February 2023 to September 2023). Clinicians helped participants complete the measurement tools to avoid the presence of missing data.

### 2.4. Statistical Analysis

Statistical analysis was performed with Stata18 for Windows (Stata-Corp, College Station, TX, USA) [27]. Firstly, the correlation matrix between the variables analyzed in the study was obtained. The clinical relevance of the correlation coefficients (R) was not based on the results of the significance tests using Pearson’s correlation model since this test provides results that are directly based on the sample size: very weak correlation coefficients (close to zero) achieve significant results (*p* < 0.05) with large samples, while correlation coefficients very far from zero achieve non-significant results (*p* > 0.05) with low samples. In this work, relevant correlations were considered for R-coefficients with effect size within the ranges moderate (|R| > 0.24) to large (|R| > 0.37) (these two thresholds are equivalent to the classical Cohen’s-*d* 0.50 and 0.80) [28].

The study of the underlying processes associated with BMI was conducted with path analysis procedure, which constitutes a straightforward extension of classical multiple regression modeling. The aim of this statistical method is to estimate the significance and magnitude of the relationships among a set of variables, including mediational links (direct and indirect effects) [29]. Path analysis in this work was developed as a case of structural equation modeling (SEM). In psychology, this method has been used for the study of the etiopathogenesis of several processes, allowing for the estimation of the magnitude and significance of the hypothesized associations into a set of variables and facilitating testing mediational links. While path analysis has been historically used to disprove a “model” that postulates potential causal relations among variables, currently this method is also employed for both exploratory and confirmatory modeling (it allows both theory testing and theory development). The rationale for the model specification (synopsized in the path-diagram) was based on the theoretical background provided by the cumulated empirical evidence. All parameters were free estimated (no values were assumed, and coefficients were directly estimated by the SEM). In this work, the maximum-likelihood estimation method was used, and multi-group processes were tested to assess invariance by participants‘ sex and diagnostic subtype. Goodness-of-fit was considered based on the usual statistical measures [30,31]: non-significant result in the chi-square (χ^2^) test, Root Mean Square Error of Approximation RMSEA < 0.08, Tucker–Lewis Index TLI > 0.90, Bentler’s Comparative Fit Index CFI > 0.90, and Standardized Root Mean Square Residual SRMR < 0.10.

## 3. Results

### 3.1. Descriptive Statistics for the Sample

The frequency distribution of the variables analyzed in the study and the comparison between the diagnostic subtypes are displayed in Table 1. Most participants in the complete sample were women (64.2%), and the mean age was 32.1 years (SD = 6.26). No differences between the diagnostic groups were found.

The correlation matrix between the variables considered in the study is displayed in Table 2. Positive coefficients were obtained (R values within the ranges mild-moderate to large-high), suggesting the relationships between difficulties with emotion regulation, higher impairment in cognitive decision-making, higher impulsivity, higher anxiety, higher stress, higher eating symptom level, and higher BMI.

### 3.2. Path Analysis

**Figure 1:**
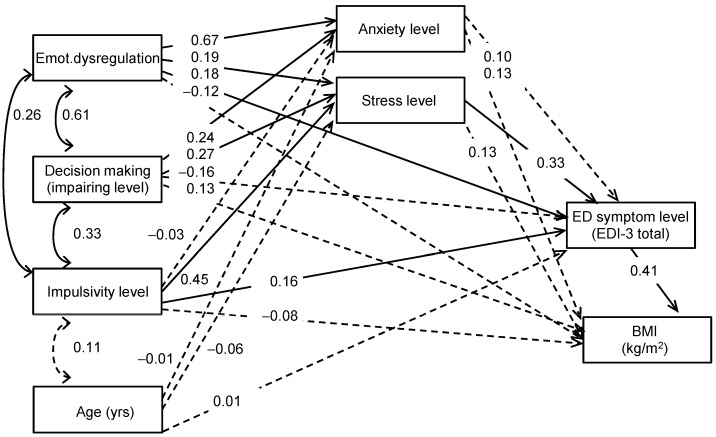
Path diagram: standardized coefficients in the complete sample. Note. Continuous line: significant parameter. Dash line: non-significant parameter.

Figure 1 shows the path diagram with the standardized coefficients obtained in the whole sample. Adequate goodness-of-fit was obtained for the SEM (see fit-indexes reported in the first line of Table 3). The results obtained in this model showed a direct effect of the EDI-3 total score on the BMI (the higher the ED symptom level, the higher the BMI). Severity of the ED was also a mediational variable within different paths: (a) difficulties with emotion regulation, higher impaired decision-making, higher impulsivity levels, increased stress levels, and higher stress levels contributed to ED with higher severity levels, which then contributed to an increased BMI; (b) higher emotion dysregulation and higher impulsivity also directly impacted severity levels of ED, which then increase BMI values. The path diagram displayed in Figure 1 also indicates that anxiety levels were directly increased for patients with difficulties with emotion regulation and decision-making.

The multi-group models defined to assess the invariance of the SEM by patients’ sex and diagnostic subtype obtained adequate goodness-of-fit (Table 3). Table 4 shows the tests for the unvarying of the structural coefficients, the covariances, and the global fit statistics. In relation to sex, join test showed invariance of the structural coefficients (the only difference was for the coefficient measuring the association between impulsivity and stress) and for the fitting indexes, but differences for the covariances emerged (two coefficients were different between sexes: impulsivity correlated with age and with decision-making only among the women’s subsample). Table S1 (Supplementary Materials) shows the structural coefficients obtained in the multi-group model defined to assess invariance by participants’ sex.

The multi-group model defined to assess the invariance of the SEM by diagnostic subtype 4 showed unvarying covariances and global fit statistics but differences in the structural coefficients. Figure 1 shows the path diagrams with the standardized coefficients obtained from each diagnosis. The test of invariance for each structural coefficient reported differences for: (a) the contribution of emotion dysregulation to the stress level (a significant coefficient was found for NES and BN, while no association was identified for OSFED and BED); (b) the effect of ED symptom severity on BMI (the relationship was identified for OSFED, NES, and BN but was not relevant for BED); and (c) the impact of decision-making on BMI (a significant association only found among the OSFED group) (see Table S2, Supplementary, for the structural coefficients obtained in the multi-group model defined, including the diagnostic subtype as a group).

## 4. Discussion

This study aimed to explore the pathways between eating related symptoms (disordered eating characteristics), emotional dysregulation, impaired decision-making, impulsiveness, anxiety, stress, and BMI, among patients diagnosed with ED (OSFED, BED, NES, and BN).

The results indicated that higher levels of symptoms of ED directly impacted the BMI (direct effect). This observation is consistent with previous studies [32] that observed the pathognomonic nature of disordered/impairing eating behaviors on the body composition (and particularly on BMI) [33]. It is well known that some ED subtypes (such as BN and BED) show symptoms related to the sensation of loss of control, a feature strongly linked with the inability to stop eating. Patients within these ED categories also report eating faster than normal (even being alone) and argue that this eating style may prevent others from realizing how much they eat (a behavior contributing to the chronification) [33,34].

Another relevant result of our study is the identification of levels of symptoms of ED as a mediational link in various pathways. In particular, difficulties with emotional regulation, greater deterioration in decision-making, higher levels of impulsivity, and increased stress levels contributed to higher levels of severe symptoms in the ED, which finally lead to higher BMI. These results are consistent with previous studies that outline the influence of emotional dysregulation [8,9,10,11], difficulties in decision-making [13,14,18,19], and impulsivity [35,36], on increased anxiety and perceived stress, which next lead to impairing/disordered eating behaviors. Our results support previous studies that suggest that stress plays an important role in the onset and development of ED [37,38,39]. Concretely, stress has been associated with an increased likelihood of being overweight/obese [40,41,42], arguing that exposure to life events may release appetite hormones that increase the desire for foods rich in fats and sugars [40] with weak efforts for healthy activity levels [43]. Studies have also observed that participants who reported a higher perception of stress were more likely to indicate a lower intake of fruits, vegetables, and proteins, but higher consumption of salty and sweet snacks and less participation in physical activities [42]. A meta-analysis of prospective studies has also revealed that psychosocial stressors, including acute stressful events (for example, being fired or losing a loved one) are risk factors for increased BMI [43]. Other forms of psychosocial stressors have also been postulated as potential risk factors for overweight and obesity in adulthood, such as weight discrimination (i.e., unfair treatment of a person based on their weight) [44], childhood trauma (e.g., physical abuse, verbal abuse, witnessed abuse, humiliation, neglect) [45], financial stress (e.g., difficulty paying bills) [46], and conflicts in interpersonal relationships (e.g., adverse exchanges and conflicts) [47].

Our study also evidenced the mediating role of the severity of the symptoms of ED on BMI levels. The pathways showed that difficulties with emotional regulation capacity and higher levels of impulsivity directly impacted the severity levels of ED, and the more impaired they were, the more severe the ED were and the higher the BMI was. This new empirical observation reinforces previous studies that concluded that both emotion regulation and impulsiveness are key features in the genesis and maintenance of disordered eating behaviors [48,49,50,51,52].

Related to the previous result, this work showed that anxiety levels were directly related to the individuals’ difficulties regulating their emotions as well as to impaired decision-making. The crucial role of negative mood states and concrete anxiety states on the etiopathogenesis of multiple mental disorders has been systematically reported [53,54]. Existing research also suggests that emotion regulation skills are transdiagnostic factors for the development and course of anxiety disorders [53,54] and also for the different subtypes of ED [54,55]. It could be that individuals with difficulties managing emotional responses and eating experiences accumulate periods of more serious stress, which can evolve into anxiety levels and subsequently to greater severity of their ED [10,33,54]. Studies have also observed that patients with poor emotional awareness reported higher anxiety levels [54,56,57], suggesting that these subjects typically avoid situations of high emotional cost because of the hard time coping with these states. That is, since emotional awareness capacities allow people to acknowledge the type of emotions and the degree to which they are experienced, the difficulties with recognizing negative emotions should increase distress tolerance, capacities for managing conflict, and impairing decision-making. All these features form the basis of the severity of ED, and therefore are mediational links for the BMI. These pathways have been suggested based on the empirical results obtained in previous studies with samples of overweight and/or obese patients [12,38,39,43,50,58] and patients with ED [59,60]. Previous studies identified the direct contribution of emotional regulation on the perceived stress levels among NES [3,61,62,63], BN [57,64,65], BED [2,4,5,18,36,37,52,58], and OSFED [66,67,68].

The association between high anxiety and impaired decision-making has also been reported in previous research analyzing the diverse endophenotypes of ED [13,14,18,19,69]. Both features have been identified as triggers for disordered eating (restrictive or compulsive eating, irregular or inflexible eating patterns), and for multiple behaviors present in people with ED (fasting, binges, skipping meals, self-induced vomiting, use of diet pills, use of laxatives-diuretics, etc.). Furthermore, the study of the neurobiology of ED has identified multifaceted interaction systems involving both neural and physical substrates for hunger and satiety, including the diverse processes implicated in eating (hedonic/reward-based homeostatic/appetitive actions) [70]. The reward circuitry includes multiple brain areas (ventral striatum, amygdala, thalamus, and midbrain neuros), as well as neurotransmitters and neuropeptides (dopamine, serotonin, opioids, endocannabinoids, orexigenic peptides, and glutamate) [71]. Studies aimed at investigating the neural correlations of decision-making processes among patients with ED have observed aberrant functioning of these brain areas [72]. For example, poor cognitive flexibility in AN seems characterized by impaired functioning of the frontoparietal control network, and this weakened performance may contribute to dysregulation in goal-directed cognition and decision-making [73]. The difficulties with cognitive performance in BN patients have been associated with premotor cortex and dorsal striatum [65]. Etiological studies have also associated high anxiety levels and body shape concerns among AN patients with abnormal brain functioning, concretely with prefrontal and cingulate brain response [70,74,75], and with lower caudate or parietal activity [75], which are also strongly related with cognition processes. But these studies should be considered with caution, despite the number of studies aimed at identifying brain structures and structural connectivity contributing to AN, BN, and OB (such as food restriction, binge-eating or purging behaviors, cognitive and emotional factors), empirical research is still low for BED.

Regarding the differences by sex, our study showed invariance for the SEM. This result seems inconsistent with previous studies which observed differences between men and women in most of the variables analyzed in this work (female sex were related to higher impulsivity levels, more perceived stress, more difficulties in decision-making, and higher severity of ED) [59,60,76,77,78]. Regarding this supposed discrepancy, it should be considered that our study did not analyze mean differences between men and women in these constructs, but rather assessed whether the underlying relationships (the structural coefficients) were similar. For example, our objective was not to identify differences in the symptom levels of ED between the sexes, but to test the contribution of the severity of ED on BMI (measuring the direct and indirect effects).

Similarly, we obtained invariance based on the subtype of the ED. Previous research has also outlined differences within the different groups of ED, even between BED and obese patients [59]. In fact, given the high concurrence between these diseases, both clinical conditions have some shared behaviors and features (overeating and high BMI), and etiology and behaviors were expected to have a similar presentation. However, emerging studies evidence divergent neurobiological [79,80] and psychological processes, implied in impulsivity/compulsivity response, reward processing and emotional reactivity. Our study has evidenced structural invariance related with the subtypes of ED, suggesting that the relationships pattern and the strengths of the associations did not statistically differ between the ED groups. That is, the underlying relationships between the variables analyzed in the study work in the same way across the different types of ED.

There are several limitations in the present study. First, while structural invariance was analyzed by the subtypes of the ED, no differentiation was made within each group of ED (for example, differentiating between BN-restrictive and BN-purgative). Second, comorbidities associated with eating disorders were not considered (such as diseases related to obesity, joint problems, heart disease, type 2 diabetes, gastroesophageal reflux disease, and some sleep-related breathing disorders). Finally, the sample size could be considered too small to achieve statistical power for path-analysis. However, it must be considered that the sample size requirements for these models seem to rely on outdated rules-of-thumb. Some current studies have been analyzed through Monte-Carlo procedures where the sample size requirements for some common types of SEMs is different, including variation by the number of factors, number of indicators, strength of the indicator loadings and the regressive paths and the amount of missing data per indicator [81,82]. For example, the study of Wolf and colleagues, performed with respect to statistical power, bias in the parameter estimates, and overall solution propriety, revealed that the sample requirements were into a very broad range (from 30 to 460), depending on the analysis characteristics [83]. The most interesting finding is that overall, solutions that met fitting at a given sample size were stable relative to the results of the analysis at the next largest sample sizes. Other studies have also observed that the appropriate sample size for a SEM depends on the complexity of the model, such as the following: (a) the number of parameters (at least 10 cases for each free parameter in the model [84]; (b) the complexity of the path diagram (200 to 500 for models without latent variables, and 500–1000 for models with latent variables) [85].

This study has various strengths. First the inclusion of multiple variables contributing to impaired eating behaviors, eating symptom severity, and BMI. While several previous studies analyzed the direct impact of multiple psychological features on the BMI among patients who met different diagnoses of ED, to our knowledge, this is the first study to include a broad set of indicators in a mediational model (path analysis) that allows measurement of both direct and indirect effects. This work has modeled the underlying processes between emotion regulation, impulsiveness, negative emotions (anxiety and stress), performance on decision-making, symptom severity of ED, and BMI. For example, while previous studies identified relationships between difficulties in emotion regulation strategies and impaired decision-making with BMI, our study showed that the specific contribution of these variables is explained through the mediation of stress levels and severity of the ED. The second strength is the use of path analysis implemented through SEM. This analytical approach has allowed testing the invariance of the structural network/coefficients by the patients’ sex and the subtypes of the ED. In etiological research, SEM has proved to be very useful for modeling complex clinical profiles (instead of focusing on bivariate associations between the variables), with the aim of visualizing the pattern of relationships and identifying both direct and indirect effects.

## 5. Conclusions

The results obtained in this work may contribute to the development of prevention and early detection plans, aimed at highly vulnerable populations characterized by neuropsychiatric risk and associated factors for ED (such as high impulsivity, negative mood states, and emotion dysregulation). Also, for the planning of more effective treatments for ED (concretely for BN, BED, NES, and OSFED), both prevention and intervention plans should include healthy dietary patterns and other healthy daily habits (such as adequate sleep and physical activity routines). These lifestyles can promote adequate brain health and cognitive performance, which also improve psychological well-being.

The empirical evidence of this work can also contribute to improve current psychological interventions for ED with neuronutrition research, a new scientific field that includes a subset of nutritional neurosciences to improving eating behavior, mental health and cognitive functions (in healthy and sick individuals) [86]. Reinforced psychotherapy with specific neuronutrients depending on the specific neuronutritional target may be promising multidisciplinary approach to brain health and treatment of psychiatric disorders.

Finally, we consider that the results of our study provide implications for health policy, creating a specific starting point for implementing prevention programs that jointly address mental health and eating habits, promoting early intervention on increased BMI in patients diagnosed with a subtype of ED. Health policy makers and health systems could implement more comprehensive treatments by prioritizing interdisciplinary care models, with greater investment in combined mental health and nutrition services. Meanwhile, regarding implications for clinical practice, treatments could be tailored to the individual needs of patients, considering both their psychoneurological and nutritional profile. In addition, health professionals could identify signs of psychoneurological factors that predispose and act as mediators in increased BMI in patients with ED, allowing early interventions and reducing public health costs.

## Figures and Tables

**Table 1:** Descriptive statistics for the variables of the study by diagnostic subtype group.

	OSFED (*n* = 83)		BED (*n* = 37)		NES (*n* = 31)		BN (*n* = 42)			
	*n*	%	*n*	%	*n*	%	*n*	%	χ^2^	*p*
Sex										
Women	52	62.7%	23	62.2%	22	71.0%	27	64.3%	0.77	0.856
Men	31	37.3%	14	37.8%	9	29.0%	15	35.7%		
	Mean	SD	Mean	SD	Mean	SD	Mean	SD	F-stat	*p*
Age	32.20	6.21	32.38	6.61	32.16	6.21	31.76	6.31	0.071	0.975
Emotion dysregulation	12.47	4.35	12.95	4.48	13.55	5.62	12.95	4.04	0.452	0.716
Decision-making	12.93	5.96	14.32	6.58	14.00	5.68	14.60	6.12	0.905	0.440
Impulsivity	14.45	4.28	14.81	4.91	14.65	4.82	14.79	5.01	0.077	0.972
Anxiety	14.39	4.90	15.16	5.75	15.77	6.06	15.52	5.52	0.713	0.545
Stress	22.70	7.19	24.70	9.05	24.74	9.67	24.00	8.47	0.769	0.513
Eating symptom level	4.93	2.13	4.95	2.20	5.68	3.32	5.31	2.73	0.815	0.487
BMI (kg/m^2^)	29.35	10.73	27.84	7.37	30.65	13.81	30.93	13.27	0.590	0.622

Note. SD: standard deviation. OSFED: other specified feed and eating disorder. BED: binge eating disorder. NES: night eating syndrome. BN: bulimia nervosa. Comparison between the groups through chi-square (χ^2^) for categorical variables and analysis of variance (ANOVA) for quantitative variables.

**Table 2:** Correlation matrix.

		2	3	4	5	6	7	8	9
1	Age	0.030	0.110	0.111	0.029	0.026	0.017	0.052	−0.080
2	Emotion dysregulation		**0.607** ^ † ^	**0.262** ^ † ^	**0.803** ^ † ^	**0.467** ^ † ^	**0.356** ^ † ^	**0.245** ^ † ^	−0.105
3	Decision-making			**0.333** ^ † ^	**0.632** ^ † ^	**0.525** ^ † ^	0.234	**0.277** ^ † ^	−0.133
4	Impulsivity				0.221	**0.586** ^ † ^	**0.366** ^ † ^	0.187	0.031
5	Anxiety					**0.445** ^ † ^	**0.321** ^ † ^	**0.284** ^ † ^	−0.117
6	Stress						**0.467** ^ † ^	**0.345** ^ † ^	−0.114
7	Eating symptom level							**0.468** ^ † ^	−0.010
8	BMI (kg/m^2^)								0.010
9	Sex								---

Note. ^†^ Bold: effect size within the ranges mild–moderate to large–high.

**Table 3:** Fit statistics for the SEM.

	χ^2^	*p*	RMSEA	CFI	TLI	SRMR
Single	1.220	0.544	0.001	0.999	0.999	0.008
Invariance by sex	0.885	0.927	0.001	0.999	0.999	0.008
Invariance by diagnostic subtype	4.903	0.768	0.001	0.999	0.999	0.019

Note. SD: standard deviation. RMSEA: Root mean squared error of approximation. CFI: Comparative fit index. TLI: Tucker–Lewis index. SRMR: Standardized root mean squared residual.

**Table 4:** Tests for group invariance.

		Sex				Diagnostic Subtype			
Structural Coeff.		χ^2^		*df*	*p*	χ^2^		*df*	*p*
Stress	Age	0.022		1	0.881	4.963		3	0.175
	Impulsivity	8.419		1	0.004 *	2.730		3	0.435
	decision-making	1.949		1	0.163	4.695		3	0.196
	Emotion dysregulation	2.518		1	0.113	8.183		3	0.042 *
Anxiety	Age	0.002		1	0.968	5.067		3	0.167
	Impulsivity	0.172		1	0.678	3.001		3	0.392
	decision-making	1.263		1	0.261	5.436		3	0.143
	Emotion dysregulation	0.711		1	0.399	1.350		3	0.717
Symptoms of ED	Stress	1.774		1	0.183	0.344		3	0.952
	Anxiety	1.383		1	0.240	5.405		3	0.144
	Age	1.240		1	0.266	5.752		3	0.124
	Impulsivity	0.723		1	0.395	6.920		3	0.075
	decision-making	0.023		1	0.880	5.978		3	0.113
	Emotion dysregulation	1.209		1	0.272	1.485		3	0.686
BMI	Stress	0.744		1	0.388	3.759		3	0.289
	Anxiety	0.008		1	0.929	1.805		3	0.614
	ED symptom level	2.588		1	0.108	12.767		3	0.005 *
	Impulsivity	0.164		1	0.685	4.717		3	0.194
	decision-making	0.026		1	0.872	8.011		3	0.046 *
	Emotion dysregulation	0.634		1	0.426	4.119		3	0.249
Join tests	Parameters		χ^2^	*df*	*p*		χ^2^	*df*	*p*
	Structural coefficients		24.940	20	0.204		108.873	60	0.001 *
	Covariances		18.610	10	0.045 *		22.520	30	0.835
	Fit-statistics	Women	0.432	2	0.806	OSFED	0.296	2	0.862
		Men	0.453	2	0.797	BED	1.172	2	0.557
						NES	1.850	2	0.397
						BN	1.584	2	0.453

Note. *df*: degrees of freedom. OSFED: other specified feed and eating disorder. BED: binge eating disorder. NES: night eating syndrome. BN: bulimia nervosa. * Bold: significant test.

## Data Availability

The raw data supporting the conclusions of this article will be made available by the authors, without undue reservation.

## Supplementary Materials

The following supporting information can be downloaded at: https://www.mdpi.com/article/10.3390/nu17020296/s1, Table S1: Structural coefficients in the multi-group model including the sex as a group; Table S2: Structural coefficients in the multi-group model including the diagnostic subtype as a group.
