# Supplementary material for: Psychoneurological Links Contributing to Body Mass Index and Eating Disorder Severity
Source: Nutrients. 2025 Jan 15;17(2):296. doi: 10.3390/nu17020296 (PMC11767959; doi:10.3390/nu17020296)
Supplement: Supplementary file 1 [file nutrients-17-00296-s001.zip › nutrients-3363630-supplementary.pdf]

Table S1 (supplementary material) Structural coefficients in the multi-group model including the sex as a group

		Women			Men		
		Coeff.	*SE*	*p*	*Coeff.*	*SE*	*p*
Stress	Age	-0.087	0.060	0.148	-0.083	0.097	0.394
	Impulsivity	0.551	0.059	<0.001	0.322	0.097	0.001
	Decision making	0.173	0.076	0.022	0.407	0.128	0.002
	Emotion dysregulation	0.242	0.071	0.001	0.052	0.136	0.705
Anxiety	Age	-0.020	0.054	0.711	-0.016	0.063	0.801
	Impulsivity	-0.011	0.059	0.853	-0.048	0.065	0.455
	Decision making	0.209	0.068	0.002	0.307	0.086	<0.001
	Emotion dysregulation	0.674	0.055	<0.001	0.627	0.081	<0.001
ED level	Stress	0.283	0.116	0.015	0.437	0.115	<0.001
	Anxiety	0.169	0.130	0.194	-0.102	0.188	0.588
	Age	0.050	0.079	0.532	-0.091	0.099	0.355
	Impulsivity	0.196	0.106	0.066	0.070	0.109	0.523
	Decision making	-0.177	0.104	0.090	-0.129	0.154	0.403
	Emotion dysregulation	0.109	0.131	0.403	0.366	0.179	0.041
BMI	Stress	0.095	0.117	0.419	0.258	0.142	0.069
	Anxiety	0.109	0.129	0.399	0.112	0.203	0.583
	ED symptom level	0.448	0.081	<0.001	0.293	0.126	0.020
	Impulsivity	-0.097	0.104	0.351	-0.054	0.118	0.649
	Decision making	0.138	0.103	0.180	0.186	0.164	0.257
	Emotion dysregulation	-0.048	0.128	0.707	-0.274	0.200	0.170
Covar.	Age - Impulsivity	0.184	0.087	0.034	-0.041	0.120	0.735
	Age - Decision making	0.125	0.088	0.156	0.038	0.120	0.753
	Age - Emotion dysregul.	0.072	0.089	0.418	-0.088	0.119	0.461
	Impulsivity - Decision making	0.407	0.075	<0.001	0.187	0.116	0.107
	Impulsivity - Emotion dysregul.	0.260	0.084	0.002	0.282	0.111	0.011
	Decision making - Emotion Dysregul.	0.568	0.061	<0.001	0.686	0.064	<0.001

Note. Coeff: standardized coefficient. SE: standard error. Covar: covariances

Table S2 (supplementary material) Structural coefficients in the multi-group model including the diagnostic subtype as a group

		OSFED			*BED*			*NES*			*BN*		
		Coeff.	*SE*	*p*	*Coeff.*	*SE*	*p*	*Coeff.*	*SE*	*p*	*Coeff.*	*SE*	*p*
Stress	Age	-0.201	0.090	0.026	-0.112	0.107	0.297	0.054	0.103	0.601	0.072	0.105	0.490
	Impulsivity	0.391	0.087	<0.001	0.506	0.109	<0.001	0.538	0.108	<0.001	0.456	0.117	<0.001
	Decision making	0.354	0.109	0.001	0.527	0.157	0.001	0.037	0.129	0.775	0.238	0.135	0.077
	Emotion dysregulation	0.071	0.111	0.521	-0.132	0.152	0.385	0.405	0.140	0.004	0.270	0.118	0.022
Anxiety	Age	-0.077	0.070	0.269	0.065	0.068	0.340	0.114	0.091	0.213	-0.114	0.099	0.250
	Impulsivity	0.033	0.070	0.638	-0.096	0.074	0.195	-0.113	0.102	0.266	-0.155	0.116	0.183
	Decision making	0.174	0.086	0.043	0.441	0.102	<0.001	0.119	0.113	0.291	0.304	0.128	0.017
	Emotion dysregulation	0.665	0.073	<0.001	0.584	0.093	<0.001	0.842	0.103	<0.001	0.642	0.097	<0.001
ED level	Stress	0.378	0.105	<0.001	0.467	0.228	0.041	0.317	0.232	0.172	0.223	0.193	0.249
	Anxiety	0.050	0.142	0.727	0.043	0.375	0.910	0.642	0.261	0.014	-0.089	0.207	0.666
	Age	0.189	0.093	0.042	-0.193	0.156	0.215	-0.055	0.135	0.683	-0.077	0.135	0.567
	Impulsivity	0.179	0.099	0.072	-0.326	0.206	0.113	0.230	0.196	0.240	0.317	0.178	0.075
	Decision making	-0.156	0.120	0.195	0.054	0.303	0.859	-0.477	0.167	0.004	-0.015	0.188	0.935
	Emotion dysregulation	0.269	0.145	0.063	0.095	0.312	0.761	-0.130	0.290	0.654	0.210	0.205	0.306
BMI	Stress	0.148	0.117	0.204	0.388	0.251	0.122	0.258	0.254	0.310	-0.179	0.169	0.289
	Anxiety	0.299	0.145	0.040	0.465	0.379	0.220	-0.050	0.304	0.870	0.046	0.176	0.794
	ED symptom level	0.391	0.107	<0.001	0.096	0.166	0.562	0.418	0.185	0.024	0.685	0.114	0.001
	Impulsivity	-0.194	0.103	0.059	0.139	0.220	0.527	0.135	0.212	0.525	-0.242	0.159	0.127
	Decision making	0.264	0.121	0.030	-0.516	0.303	0.088	0.276	0.198	0.164	0.235	0.161	0.145
	Emotion dysregulation	-0.379	0.152	0.013	-0.274	0.309	0.375	-0.168	0.295	0.569	0.142	0.176	0.419
Covar.	Age - Impulsivity	0.211	0.105	0.044	0.184	0.159	0.247	0.171	0.174	0.326	-0.161	0.150	0.283
	Age - Decision making	0.135	0.108	0.210	0.202	0.158	0.200	0.079	0.178	0.656	0.004	0.154	0.977
	Age - Emotion dysregul.	0.032	0.110	0.770	-0.010	0.164	0.953	-0.056	0.179	0.754	0.155	0.151	0.302
	Impulsivity - Decision making	0.153	0.107	0.154	0.478	0.127	<0.001	0.275	0.166	0.097	0.535	0.110	<0.001
	Impulsivity - Emotion dysregul.	0.132	0.108	0.221	0.375	0.141	0.008	0.465	0.141	0.001	0.202	0.148	0.172
	Decision making - Emotion dysregul.	0.606	0.070	<0.001	0.728	0.077	<0.001	0.627	0.109	<0.001	0.494	0.117	<0.001

Note. Coeff: standardized coefficient. SE: standard error. Covar: covariances.

OSFED: other specified feed and eating disorder. BED: binge eating disorder.

NES: night eating syndrome. BN: bulimia nervosa.
